# Ultrasensitive Imaging of Ca^2+^ Dynamics in Pancreatic Acinar Cells of Yellow Cameleon-Nano Transgenic Mice

**DOI:** 10.3390/ijms151119971

**Published:** 2014-11-03

**Authors:** Yusuke Oshima, Takeshi Imamura, Atsuko Shintani, Hiroko Kajiura-Kobayashi, Terumasa Hibi, Takeharu Nagai, Shigenori Nonaka, Tomomi Nemoto

**Affiliations:** 1 Molecular Medicine for Pathogenesis, Graduate School of Medicine, Ehime University, Toon City, Ehime 791-0295, Japan; E-Mail: timamura-ind@umin.ac.jp; 2 Division of Bio-Imaging, Proteo-Science Center, Ehime University, Toon City, Ehime 791-0295, Japan; 3 Translational Research Center, Ehime University Hospital, Toon City, Ehime 791-0295, Japan; 4 Laboratory for Spatiotemporal Regulations, National Institute for Basic Biology, Okazaki, Aichi 444-8585, Japan; E-Mails: ashin@ims.ac.jp (A.S.); hiroko@nibb.ac.jp (H.K.-K.); snonaka@nibb.ac.jp (S.N.); 5 Laboratory of Molecular and Cellular Biophysics, Research Institute for Electronic Science, Hokkaido University, Sapporo, Hokkaido 001-0020, Japan; E-Mail: thibi@es.hokudai.ac.jp; 6 The Institute of Scientific and Industrial Research, Osaka University, Ibaraki, Osaka 567-0047, Japan; E-Mail: ng1@sanken.osaka-u.ac.jp

**Keywords:** Yellow Cameleon, Ca^2+^ indicator, Ca^2+^ imaging, Förster resonance energy transfer (FRET), genetically encoded Ca^2+^ indicators (GECI), Yellow Cameleon-Nano (YC-Nano), two-photon excitation fluorescence microscopy, live-cell imaging, exocytotsis, acinar cell

## Abstract

Yellow Cameleons are genetically encoded Ca^2+^ indicators in which cyan and yellow fluorescent proteins and calmodulin work together as a fluorescence (Förster) resonance energy transfer Ca^2+^-sensor probe. To achieve ultrasensitive Ca^2+^ imaging for low resting Ca^2+^ or small Ca^2+^ transients in various organs, we generated a transgenic mouse line expressing the highest-sensitive genetically encoded Ca^2+^ indicator (Yellow Cameleon-Nano 15) in the whole body. We then focused on the mechanism of exocytotic events mediated by intracellular Ca^2+^ signaling in acinar cells of the mice with an agonist and observed them by two-photon excitation microscopy. In the results, two-photon excitation imaging of Yellow Cameleon-Nano 15 successfully visualized intracellular Ca^2+^ concentration under stimulation with the agonist at nanomolar levels. This is the first demonstration for application of genetically encoded Ca^2+^ indicators to pancreatic acinar cells. We also simultaneously observed exocytotic events and an intracellular Ca^2+^ concentration under *in vivo* condition. Yellow Cameleon-Nano 15 mice are healthy and no significant deteriorative effect was observed on physiological response regarding the pancreatic acinar cells. The dynamic range of 165% was calculated from *R*_max_ and *R*_min_ values under *in vivo* condition. The mice will be useful for ultrasensitive Ca^2+^ imaging *in vivo*.

## 1. Introduction

Ca^2+^ signaling plays crucial roles in regulating a wide variety of physiological processes. The up-regulation of intracellular free Ca^2+^ is mediated by release of Ca^2+^ from intracellular stores, a process known as inositol triphosphate-induced Ca^2+^ release (IICR) or Ca^2+^-induced Ca^2+^ release (CICR), as well as by voltage-dependent Ca^2+^ influx from the extracellular fluid. Exocytosis, in which the contents of secretory vesicles are released, is very important in cytophysiology [1]. Exocytotic secretion in pancreatic acinar cells is mediated by cholecystokinin (CCK) or acetylcholine (ACh) as a result of increases in the cytosolic concentration of Ca^2+^ [2]. Two-photon excitation microscopy is a powerful tool for observing intracellular Ca^2+^ and exocytotic events simultaneously *in vitro*. Ca^2+^-dependent exocytosis has been visualized by two-photon excitation imaging in pancreatic acinar cells [3].

Cytosolic and organellar free Ca^2+^ concentrations can be measured *in vitro* by using fluorescence microscopy to detect synthetic fluorescent dyes [4,5,6], but it is difficult to precisely target these dyes to specific intracellular locations [7,8], and there are also limits to the cell-type specificity that can be achieved [9]. Furthermore, these extrinsic indicators are not applicable to *in vivo* analysis, due to the invasiveness of the procedures required to introduce them [8]. To address this issue, in 1997 Miyawaki *et al.* developed genetically encoded Ca^2+^ indicators (GECIs) [7]. Yellow Cameleons (YCs) are widely used GECIs in which cyan and yellow fluorescent proteins (CFP and YFP) and calmodulin (CaM) work together as a fluorescence (Förster) resonance energy transfer (FRET) Ca^2+^-sensor probe. YCs are useful in live-cell Ca^2+^ imaging, but they are not suitable for observation in some cells with low resting [Ca^2+^] or small [Ca^2+^] transients less than 100 nM [10,11]. Therefore, higher-affinity indicators with smaller dissociation constant (*K_d_*) values than those of conventional GECIs are needed.

Improvements in the *K_d_* and dynamic range of GECIs have recently been reported [11,12]. For example, YC2.60 and YC3.60 have high Ca^2+^ affinities and large dynamic ranges. Horikawa *et al.* [11] described the Yellow Cameleon-Nano (YC-Nano) series, in which the linker peptide in the Ca^2+^ binding domain is elongated; these ultrasensitive GECIs have the highest affinity reported to date (15–140 nM). Those authors showed that transiently expressed YC-Nano is useful for detecting small intracellular [Ca^2+^] transients in embryos of zebrafish and in mouse neural cells following *in utero* electroporation [9,11,13]. Although the YC-nano series are useful for Ca^2+^ imaging in mammals, it is difficult to manipulate stable expression of GECIs in target tissues by transient expression.

In this study, we generated healthy transgenic mouse lines ubiquitously expressing YC-Nano 15, the highest-affinity GECI. Exocrine acinar cells have been studied as a classical model for the mechanism of exocytotic events mediated by intracellular Ca^2+^ signaling [1,3]; we chose to use this system to demonstrate the usefulness of these new mouse strains. Specifically, we performed FRET ratio imaging by two-photon microscopy of acinar cells expressing YC-Nano15 in order to visualize Ca^2+^ dynamics during agonist stimulation.

## 2. Results and Discussion

### 2.1. Ubiquitous Expression of YC-Nano15 in Transgenic Mouse Lines

**Figure 1:**
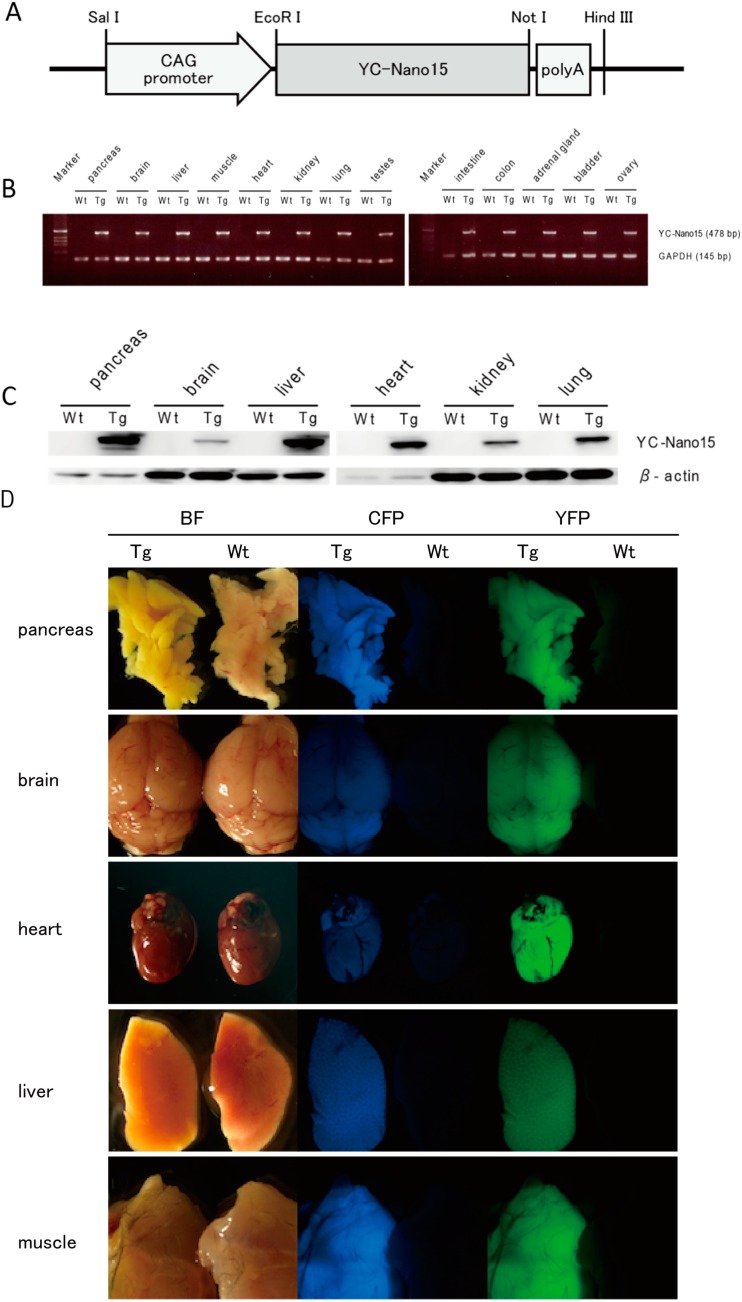
Yellow Cameleon-Nano 15 (YC-Nano15) is expressed ubiquitously in adult organs. (**A**) The YC-Nano15 transgene construct contains a 1.9-kb YC-Nano15 sequence downstream of the CAG promoter and upstream of the SV40 polyadenylation sequence; (**B**) Real time-PCR (RT-PCR) analysis of various tissues of YC-Nano15-205 mice revealed that mRNA of YC-Nano15 was expressed ubiquitously in the adult organs. In wild-type (C57BL/6J) mice, no band appeared in a PCR amplification using the YC-Nano15 primers (internal standard: *mGAPDH*); (**C**) Western-blot analysis. YC-Nano15 protein is expressed ubiquitously in adult organs; and (**D**) Fluorescence of various organs in a YC-Nano15-205 mouse. Bright-field (**left**), CFP fluorescence (**middle**), and YFP fluorescence images (**right**) of pancreas, brain, heart, liver, and muscle of a YC-Nano15-205 (Tg) mouse and a C57BL6J (wild-type) mouse are shown. The tissues of YC-Nano15-205 mice are yellower than those of wild-type mice due to expression of YC-Nano15.

We established three transgenic strains (YC-Nano15-202, -204, and -205) that express YC-Nano15 under the control of the CAG promoter (Figure 1A). All of the strains looked healthy and exhibited no apparent abnormalities relative to wild-type siblings. The YC-Nano15-205 mice were used in subsequent studies, because the fluorescence in pancreatic tissue of this strain was the brightest under fluorescence stereomicroscope observation. To determine suitability for Ca^2+^ imaging, expression levels of YC-Nano15 in various tissues of YC-Nano15-205 mice were tested by real time-PCR (RT-PCR) and western-blot analyses. Both mRNA and protein of YC-Nano15 mice were ubiquitously expressed in the adult organs of YC-Nano15-205 mice (Figure 1B,C). We examined the tissue morphology and fluorescence signals in various organs of YC-Nano15-205 mice by fluorescence stereomicroscopic observation of autopsy samples (Figure 1D), and found that size and morphology were not distinguishable between YC-Nano15-205 and wild-type mice. Fluorescence of CFP and YFP was observed in all organs examined, including pancreas, brain, heart, liver, and muscles. Because high-affinity GECIs can strongly chelate intracellular Ca^2+^, it is possible that YC-Nano15 could affect the homeostatic balance of intracellular [Ca^2+^]. However, the results of the examinations described above suggested that expression of YC-Nano15 does not affect reproductive, developmental, or other important physiological processes.

### 2.2. Evaluation of Dynamic Range of YC-Nano15 as an Ultrasensitive Ca^2+^ Indicator by Two-Photon Excitation FRET Ratio Imaging

**Figure 2:**
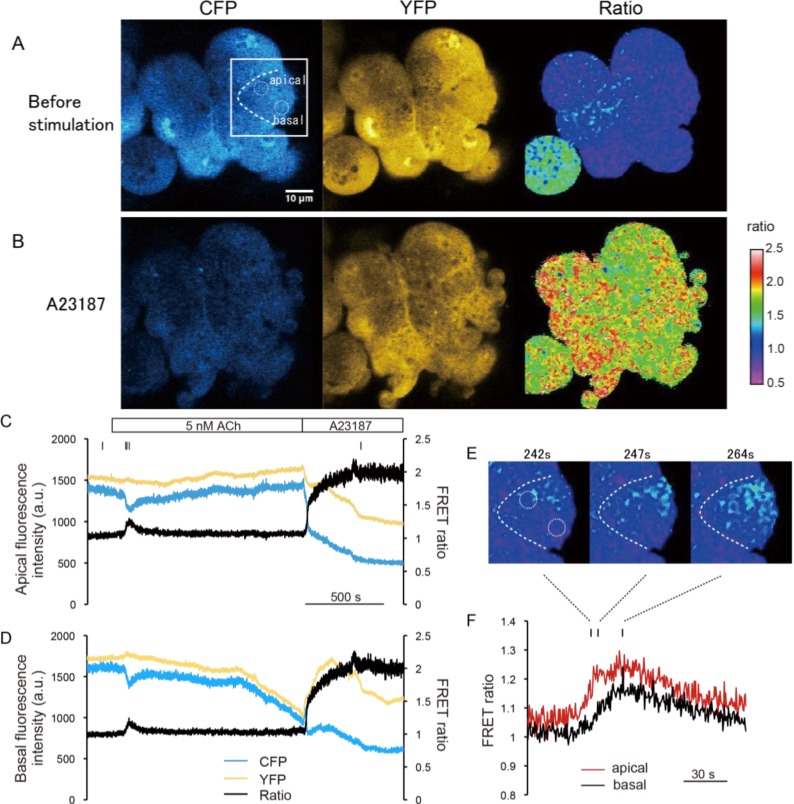
Dynamic range of the fluorescence ratio of cyan and yellow fluorescent proteins (CFP and YFP) (Förster resonance energy transfer ratio, FRET ratio) in pancreas acinar cells of YC-Nano15-205 mice. (**A**,**B**) Change in FRET signal following ionophore application. Two-photon excited fluorescence images of CFP and YFP, and fluorescence resonance energy transfer (FRET) ratio (YFP/CFP) image (right) before (**A**, −57 s) and after (**B**, 1557 s) stimulation with Ca^2+^/A23187 solution are shown; these timings are also indicated as small bars at the top of (**C**). In both (**A**) and (**B**), the FRET ratio is distributed homogenously in the cell; (**C**,**D**) Time course of CFP and YFP signals and FRET ratio in the “apical” and “basal” circles in the cell shown inside withe box in (**A**). After stimulation with a minimum concentration of 5 nM acetylcholine (ACh), the FRET ratio increased slightly; (**E**,**F**) Detailed view of FRET signal changes upon stimulation of 5 nM ACh in the same experimental trial shown in (**A**–**D**). Timings of the images in (**E**) correspond to the small bars at the top of (**F**). Unidirectional propagation of the [Ca^2+^] increase from the apical to the basal region of the cell is shown in (**E**); and (**G**) Time course of changes in the FRET ratio in the apical and basal regions of the cell shown in (**A**).

Using two-photon excitation microscopy, we performed FRET ratio imaging of pancreatic acinar cells of YC-Nano15-205 mice. Two-photon excitation microscopy can visualize deep regions of tissues and excite multiple fluorescent proteins simultaneously [3,14,15,16]. To perform the quantitative analysis of Ca^2+^ dynamics induced by the agonist stimulation, we employed ratiometric FRET imaging, and the acceptor/donor (YFP/CFP) ratio value was normalized to be 1 under normal conditions (before stimulation). First, we collected two-photon excitation fluorescence images of pancreatic acini under superfusing Solution A buffer in the absence of stimulation (Figure 2A). Both CFP and YFP signal were detected at an excitation wavelength of 850 nm. For spatiotemporal FRET analysis, we calculated FRET ratio (YFP/CFP) images. After we increased the intracellular [Ca^2+^] to 1 mM by treatment with a Ca^2+^ ionophore, the FRET ratio increased as expected (Figure 2B). Under these conditions, the morphology of these cells was significantly altered (e.g., by blebbing) probably due to the cytotoxicity of the Ca^2+^ ionophore.

Next, we carried out a time-course analysis in the apical and basal regions of cells (Figure 2C–F). In this experiment, acinar cells were exposed to agonist solution containing 5 nM ACh, followed by an ionophore solution containing 1 mM [Ca^2+^]. The FRET ratio transiently increased just after the ACh stimulus (initial peak), and settled to a slightly higher level than before the stimulus (sustained plateau) in both apical and the basal regions (Figure 2C,D). In Figure 2C,D, YFP signal slightly changed reciprocally to the donor signal (CFP). Theoretically, FRET can be quantified solely from either donor or acceptor signals, but practically retiometric analysis contrasting changes in both donor and acceptor signals are useful to exclude the possibility that the signal changes are caused by changes in concentration of the FRET probe or by drift in the focal plane. In this case, since the resting [Ca^2+^] estimated to be 100 nM is relatively higher than *K_d_* value of YC-Nano (15 nM), the dynamic range of CFP and/or YFP signal intensity is thought to be small in compared to *in vitro* condition [3]. The onset of the initial peak was slightly faster in the apical region, *i.e.*, the increase of [Ca^2+^] originated in the apical region and propagated to the basal region (Figure 2E,F). This result is consistent with a previous report that ACh stimulation induces the formation of apicobasal Ca^2+^ waves in acini of pancreatic acinar cells [17] and of guinea pig nasal glands [18]. Subsequently, the cells were exposed to Ca^2+^/A23187 solution, and the FRET ratio became homogenously saturate (Figure 2C,D). The value of the saturated FRET ratio (*R*_max_) was estimated as 2.01 ± 0.08 (mean ± SE; *n* = 6 cells).

We performed another time-course analysis to determine the lower limit of the FRET ratio (*R*_min_) of YC-Nano15 (Figure 2G). In this experiment, the extracellular fluid was first replaced with a Ca^2+^-free buffer that did not affect the FRET ratio of the cell. When this fluid was replaced with Ca^2+^-free EGTA buffer containing CPA and FCCP (indicated as “EGTA”), the FRET ratio rapidly increased, and then gradually decrease to a final value of 1.63 ± 0.04 (mean ± SE; *n* = 5 cells), probably reflecting release of Ca^2+^ from intracellular storage to the cytoplasm, followed by its leakage to the outside. Next, the fluid was replaced with Ca^2+^-free BAPTA-AM buffer (indicated as “BAPTA-AM”). In this case, the FRET ratio decreased below the initial value, indicating that the membrane-permeable chelator further depleted intracellular Ca^2+^. After that, the fluid was replaced with a Ca^2+^-containing buffer (indicated as “1 mM Ca^2+^”), but the FRET ratio continued decreasing, probably because the effect of the accumulated intracellular chelator surpassed the influx of Ca^2+^ from outside. Finally, the fluid was replaced with Ca^2+^ ionophore in Ca^2+^-free EGTA buffer (indicated as “A23187”). In this case, since 10 μM BAPTA-AM solution was superfused constantly, Ca^2+^-free BAPTA-AM could be sufficiently maintained in the cytosol, *i.e.*, the intracellular concentration of BAPTA-AM must be enormously higher than that of YC-Nano 15, stoichemiometrically most of YC-Nano 15 could be shifted to the unbounded form. The FRET ratio then further decreased to a plateau of 0.76 ± 0.02 (mean ± SE; *n* = 6 cells), reflecting complete depletion of intracellular Ca^2+^, *i.e.*, *R*_min_ under *in vivo* conditions. This result indicates that YC-Nano15 can sense extremely low levels of intracellular Ca^2+^ in mouse tissue. Nevertheless, because the transgenic mice are healthy and the homeostatic mechanism is maintained, the expression level of YC-Nano 15 is estimated to be moderate in the acinar cells. The expression level of YC-Nano 15 is critical to discuss the capability to sense the low [Ca^2+^] *in vivo*, in addition to that, the issue of the affinity *in vivo* is also related to the discussion. We then experimentally estimated the *K_d_* value of YC-Nano *in* *vivo*, for instance, considering the ratio change by the following calibration Equation (1) [5].

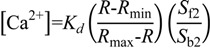
(1)

Note that *S*_f2_*/S*_b2_ is the fluorescence signal ratio of the donor channel (CFP) in free and bounded form. In this case, *S*_f2_*/S*_b2_ is not exactly-determined, but is assumed to be around 3 and over (Figure 2C,D). The *K_d_* value of YC-Nano *in* *vivo* is assumed to be to be slightly more than the resting [Ca^2+^]. On the other hand, the result also suggests that the expression level of YC-Nano is sufficient for the ratiometric analysis of physiological response to ACh stimulation in the acinar cells, but the affinity of YC-Nano 15 *in vivo* can vary tremendously according to the cell type and/or where it is expressed, probably reflecting the different degree of interaction with endogenous Ca^2+^ binding proteins in different cell types [13].

### 2.3. Spatiotemporal Analysis of Intracellular Ca^2+^ Oscillation and Exocytosis Induced by Stimulation with Agonist (ACh) in YC-Nano15-205 Mice

**Figure 3:**
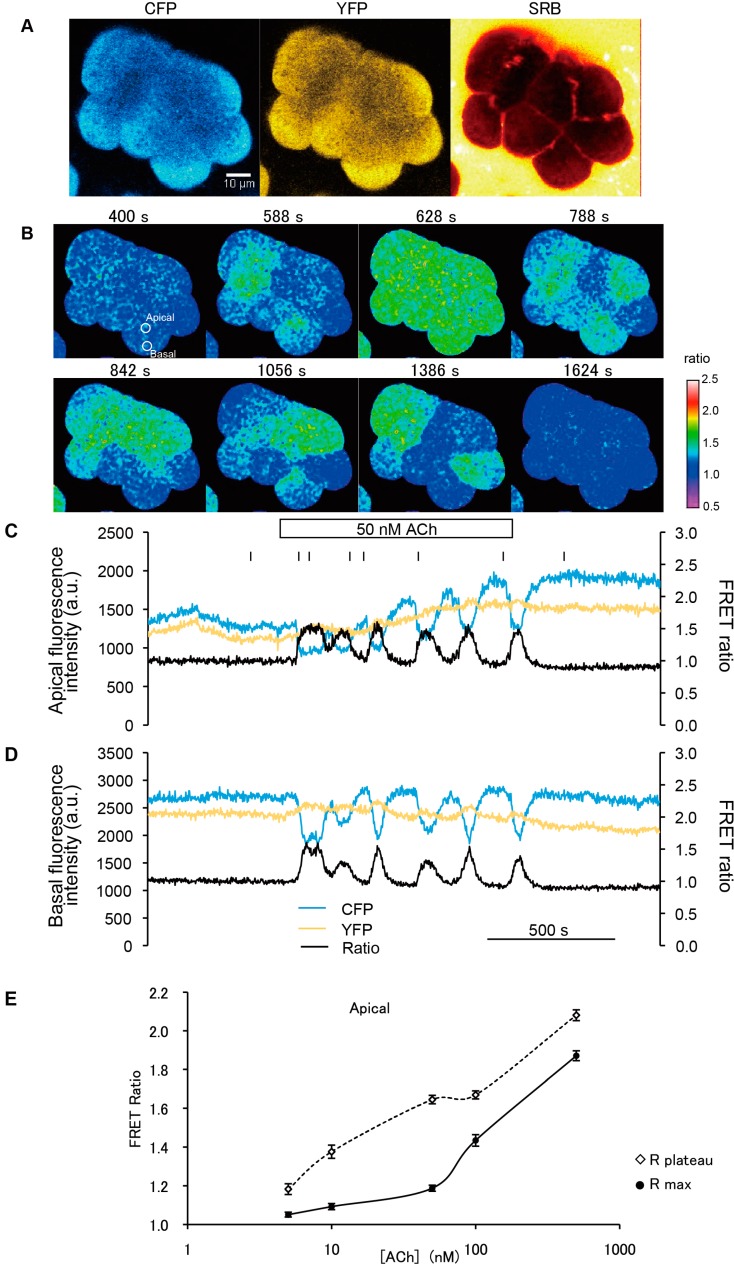
Acetylcholine (Ach)-induced Ca^2+^ oscillations revealed by FRET signals in two-photon excitation fluorescence images. (**A**) Two-photon excitation fluorescence images of CFP (**left**) and YFP (**middle**), and cross-section image in SRB solution to monitor exocytotic events (**right**), before stimulation (−144 s; time origin is the time at which the agonist was applied); (**B**) FRET images showing repeated Ca^2+^ waves from the apical to basal regions during 50 nM ACh stimulation; (**C**,**D**) Time course of changes in CFP, YFP signals, and FRET ratio in apical (**C**) and basal (**D**) regions in a cell. Regions correspond to the “apical” and “basal” circles in (**B**). Stimulation with a physiological agonist concentration of 50 nM ACh induced repeated unidirectional Ca^2+^ oscillations. After the agonist was washed out, the value of the FRET ratio returned to the initial level; and (**E**) Dose dependency of the FRET ratio on ACh concentration. In the apical region of acinar cells, the maximum peak of the FRET ratio increases as a function of acetylcholine concentration (white diamonds). FRET ratio values in sustained plateau during stimulation were plotted against the ACh concentration (black dots), although in some cases no plateau was observed (e.g., **C** and **D**, stimulated with 50 nM ACh). In those cases, FRET ratio values at the bottom of the oscillation curve were measured and plotted instead. Error bars represent S.E. The number of cells measured (*n*) at each concentration was as follows: 6 (5 nM), 17 (10 nM), 7 (50 nM), 8 (100 nM), and 10 (500 nM).

To examine both spatiotemporal Ca^2+^ dynamics and typical zymogen granule (ZG) exocytosis in pancreatic acini of YC-Nano15-205 mice, we stimulated isolated acinar cells with agonist (50 nM ACh) in sulforhodamine B (SRB) solution and observed them by two-photon excitation microscopy. Fluorescence signals of CFP, YFP, and SRB were detected simultaneously at an excitation wavelength of 850 nm (Figure 3A). An increase in intracellular [Ca^2+^] was initiated in the apical regions of acini, and [Ca^2+^] oscillated during stimulation with agonist (ACh) (Figure 3B and Supplementary Movie S1). In this analysis, the FRET ratio shuttled between the baseline and the peak values (Figure 3C,D). Exocytosis of individual secretory granules in acinar cells was evoked by this stimulation (Supplementary Movie S2). In acinar cells stimulated with 1–100 µM ACh, Ca^2+^ has been reported to transiently increase and reach a plateau [18]. However, the change in [Ca^2+^] induced by stimulation with ACh at concentrations less than 1 µM is difficult to characterize, because of the sensitivity limits of conventional synthetic Ca^2+^ indicators. In the previous report [14], both Ca^2+^ transients and exocytosis evoked by less than 50 nM ACh were observed in pancreatic acinar cells by using Fura-2FF (*K_d_* = 40 µM) and Fura-2 (*K_d_* = 200 nM) [14], but conventional synthetic Ca^2+^ indicators could not capture detailed behavior of intracellular [Ca^2+^] such as Ca^2+^ oscillation due to the limitation of the sensitivity. In this study, for the first time, we were able to successfully visualize Ca^2+^ oscillation under stimulation with agonist (ACh) at nanomolar levels in pancreatic acini of YC-Nano15 mice.

To determine the dynamic range of YC-Nano15, we also monitored the dependency of FRET ratios on ACh concentration. Acinar cells were stimulated at ACh concentrations of 5, 10, 50, 100, and 500 nM, and FRET ratios in the initial peak and subsequent plateau were calculated and plotted (Figure 3E). In the previous report by Horikawa *et al.*, the ratiometric FRET imaging of YC-Nano 15 was performed in *Dictyostlium* [11]*.* The FRET signal change (Δ*R*/*R*= (9.5 − 5.0)/5.0 × 100% = 90%) was showed *in vivo* condition. In the result, though we measured in pancreatic acinar cells of mice, the dynamic range (Δ*R/R* = (2.01 − 0.76)/0.76 × 100% = 165%) of YC-nano15 was calculated from *R*_max_ and *R*_min_ values under *in vivo* condition. The relationship between ACh concentration and FRET ratio was consistent with results described in previous reports [3,14]. Thus, the dynamic range of YC-Nano15 in mouse tissue is around 5–100 nM.

### 2.4. Imaging Analysis of Exocytotic Events in Acinar Cells

**Figure 4:**
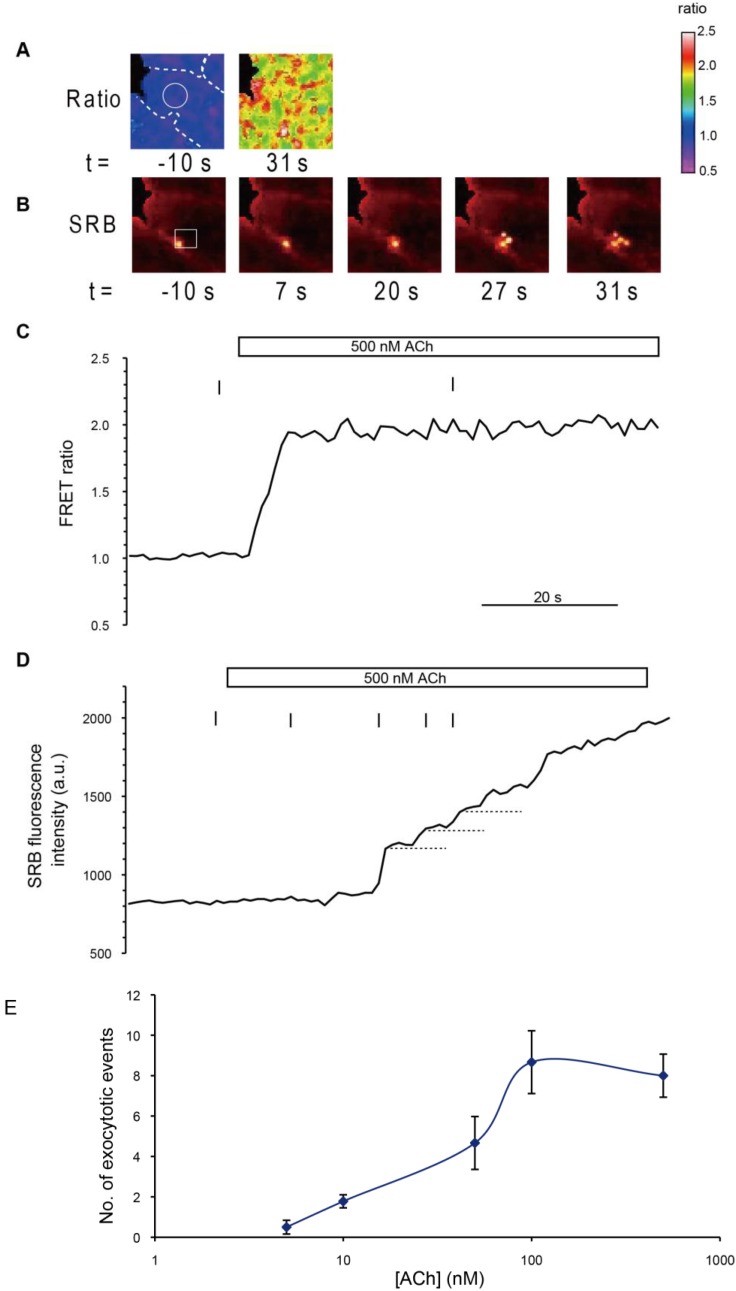
Ca^2+^-dependent sequential compound exocytosis simultaneously visualized by FRET and polar tracer signals. (**A**) FRET ratio images before (*t* = −10 s) and after (*t* = 31 s) stimulation at the time indicated by the vertical bar in (**C**); (**B**) Fluorescence images of SRB before (*t* = −10 s) and after (*t* = 7, 20, 27, and 31 s) stimulation at the time indicated by the vertical bar in (**D**); and (**C**,**D**) Time course of FRET ratio (**C**) and SRB fluorescence changes (**D**) when a high concentrations of agonist (500 nM ACh) was applied. Intensities in the white circle in (**B**) and the white box in (**C**) are plotted. The intensity of the FRET signal promptly reached the value of *R*_max_ estimated in Figure 2, indicating saturation. SRB fluorescence exhibited stepwise increases (horizontal dotted lines) simultaneous with sequential exocytotic events, shown in (**B**) (timings at −10, 7, 20, 27, and 31 s); and (**E**) The number of exocytotic events observed in each acinar cell in a 10-min interval in response to stimulation with various concentrations of ACh. The number of cells measured (*n*) at each concentration was as follows: 6 (5 nM), 9 (10 nM), 6 (50 nM), 9 (100 nM), and 13 (500 nM).

To confirm the frequency of exocytotic events induced by stimulation with agonist (ACh) at various concentrations, we performed simultaneous imaging of intracellular Ca^2+^ and exocytosis in acinar cells of YC-Nano15-205 mice, using the FRET ratio and SRB (Figure 4A,B). Stimulation with agonist (500 nM ACh) caused typical changes in the FRET ratio (Figure 4C). Individual exocytotic events were visualized, by means of the extracellular tracer SRB, as the formation of an Ω-shaped profile [14]. The fluorescence signal of SRB exhibited a stepwise increase (Figure 4D), indicating sequential exocytosis [3]. The number of exocytotic events in a 10-min interval in responses to various concentrations of ACh was also observed in each acinar cell. The results were consistent with those described in a previous report [14].

In summary, we demonstrated that ubiquitously expressed ultrasensitive GECIs are not toxic in transgenic mice, and that these reporters can be used to analyze intracellular [Ca^2+^] at physiological levels in acinar cells. The transgenic mouse strains created in this study will be useful for exploration of other previously unseen physiological phenomena at low [Ca^2+^] in tissues and in whole animals.

## 3. Materials and Methods

Main Animal experimental procedures were approved by the animal research committee of the National Institute for Basic Biology, Japan (10A091). Animals were sacrificed with cervical dislocation.

### 3.1. Generation of the YC-Nano15 Transgene

The YC-Nano15 gene was produced by digesting the pRSETb- YC-Nano15 plasmid with *Bam*HI and *Eco*RI. The 1.9-kb YC-Nano15 gene was cloned downstream of the CAG promoter, and upstream of the SV40 polyadenylation sequence, in vector pCAGGS.

### 3.2. Generation and Identification of YC-Nano15 Transgenic Mouse Lines

A 4.3-kb YC-Nano15 transgene was produced by *Sal*I-*Hin*dIII digestion of the pCAGGS-YC-Nano15 construct. This fragment was purified and microinjected into pronuclei of C57BL/6J fertilized mouse eggs, which were then transferred to pseudo-pregnant females. The transgene cloning and microinjection were performed by Unitech Co., Ltd. (Chiba, Japan). Founder mice were genotyped, using tail genomic DNA, by polymerase chain reaction (PCR) using the forward primer 5'-ACTTCAAGATCCGCCACAAC-3' and the reverse primer 5'-TTGTCAAAAACACGGAATGC-3'. Genopositive founders were backcrossed into the C57BL/6J background for production of transgenic offspring.

### 3.3. RNA Expression Analysis in Various Tissues of YC-Nano15 Mice

YC-Nano15 mice and C57BL/6J (both strains: 8–14 weeks old) were sacrificed, and organs (pancreas, intestine, colon, spleen, kidney, liver, bladder, heart, lung, brain, muscle, testes, ovary, uterus, adrenal gland) were harvested and cooled on ice. Each of the organs was washed with ice-cold PBS, cut into small pieces (3 mm × 3 mm × 3 mm), suspended in RNAlater (Qiagen, Hilden, Germany), and stored at −20 °C prior to total RNA extraction. The frozen tissues were homogenized in TRIzol Reagent (Invitrogen Corp., Carlsbad, CA, USA) using a Polytron (Kinematica, Lucerne, Switzerland) and a Dounce tissue grinder, and total RNA was isolated with chloroform and isopropanol. Precipitated total RNA was washed with 75% ethanol and redissolved in RNase-free water. Complementary DNA (cDNA) was synthesized from total RNA using the SuperScript VILO cDNA Synthesis Kit (Invitrogen Corp., Carlsbad, CA, USA). PCR was performed using cDNA template, specific primer pairs, and Taq polymerase (KAPA Taq Extra HotStart ReadyMix with dye, Nippon Genetics Co., Ltd., Tokyo, Japan) according to the recommended cycling protocol. PCR products were detected by electrophoresis assays on 2% agarose gels. The gene encoding mouse *GAPDH* (*mGAPDH*) was included to check the integrity of the RNA.

### 3.4. Western-Blotting Analysis

Various tissues were excised from YC-Nano15 mice and C57BL/6J mice (both strains: 8–14 weeks old) and immediately frozen in liquid nitrogen. To generate lysates, the tissues were homogenized in lysis buffer containing protease inhibitor, DTT, and SDS on ice. The samples were centrifuged (at 10,000 rpm, 10 min, 4 °C), and the supernatants were collected and boiled for 10 min, after which 10% SDS and beta-mercaptoethanol were added. After another centrifugation (10,000 rpm, 10 min, 4 °C), the supernatants were again collected. Proteins were separated by electrophoresis in 8% (*w*/*v*) polyacrylamide gel, and then electro-transferred onto a PVDF membrane. After blocking in TBS-T (50 mM Tris-HCl, pH 7.6, 150 mM NaCl, 0.1% Tween 20) with 5% skim milk (*w*/*v*) for 1 hour at room temperature, the membrane was incubated with anti-GFP (1:1000 dilution in 5% skim milk; M048-3; MBL, Aichi, Japan) for 1 h at room temperature. After a TBS-T rinse, the membrane was incubated with HRP-conjugated anti-goat secondary antibody (1:2000 dilution; 7076; Cell Signaling Technology, Beverly, MA, USA) for 1 h at room temperature. The membrane was then washed in TBS-T, and signals were detected by chemiluminescence (Amersham ECL Plus Western Blotting Detection System; GE Healthcare, Amersham, UK). For reblotting, membranes were stripped using Restore Western Blot stripping Buffer (Thermo Scientific, Hudson, NH, USA), followed by incubation with anti-*β*-actin serum (1:5000; AC-74; Sigma-Aldrich, St. Louis, MO, USA) for 1 h at room temperature. After a TBS-T rinse, the signal was detected by ECL.

### 3.5. Preparation of Pancreatic Acinar Cells

Clusters of acini were prepared as described in our previous reports [3,14]. In brief, clusters of acini were isolated from 8 to14-week-old mice by brief (4 min) digestion with collagenase (1 mg·mL^−1^; Wako, Osaka, Japan) followed by gentle trituration. The acini were dispersed in a small chamber and superfused (1 mL·min^−1^) with a solution termed Solution A (150 mM NaCl, 5 mM KCl, 2 mM CaCl_2_, 1 mM MgCl_2_, 10 mM HEPES-NaOH pH 7.3, and 10 mM glucose). All chemicals, except where otherwise stated, were purchased from Nacalai Tesque (Kyoto, Japan).

### 3.6. Ca^2+^ Measurement by Two-Photon Microscopy

Live-cell FRET imaging was performed using a two-photon laser scanning upright microscope (FV1000MPE, Olympus, Tokyo, Japan) with a femtosecond pulse Ti: Sapphire laser (Mai Tai HP, Spectra-physics KK, Tokyo, Japan). Acinar cells were seeded in a hand-made perfusion chamber with in and outflow channels inside a cell-culture dish (60-mm tissue-culture treated polystyrene; BD Falcon, San Diego, CA, USA), which was placed on the microscope stage. Microscopic observation was performed at room temperature. During the observation, extracellular fluid was superfused constantly, typically at a flow velocity of 0.5 mL/s. Stimulation of cells was carried out by exchanging the extracellular fluid. Acetylcholine (ACh; Sigma-Aldrich, St. Louis, MO, USA) at a concentration of 5–500 nM in Solution A was employed as a natural agonist. To increase Ca^2+^ permeability of the plasma membrane, a non-fluorescent Ca^2+^ ionophore, 4Br-A23187 (molecular probes) was first dissolved in dimethylsulfoxide (DMSO; Sigma-Aldrich, St. Louis, MO, USA) and then diluted to 10 μM in Solution A by sonication. To saturate the intercellular free Ca^2+^ concentration, 1 mM Ca^2+^ solution (140 mM NaCl, 5 mM KCl, 1 mM MgCl_2_, 10 mM HEPES-NaOH pH 7.3, and 10 mM glucose) containing 10 μM 4Br-A23187 (Ca^2+^/A23187 solution) was applied. In order to chelate intracellular or extracellular free Ca^2+^ ion, 10 mM ethylene glycol tetraacetic acid (EGTA) solution or EGTA solution containing 10 μM 4Br-A23187 (EGTA/A23187 solution) was applied. In some experiments, to chelate intracellular free Ca^2+^ we also applied 10 μM *O*,*O*'-bis(2-aminophenyl)ethyleneglycol-*N*,*N*,*N*',*N*'-tetraacetic acid, tetraacetoxymethyl ester (BAPTA-AM; Dojindo Molecular Technologies, Inc., Tokyo, Japan) in EGTA-solution (BAPTA-AM solution).

An excitation wavelength of 850 nm was used for YFP–CFP FRET imaging and cross-section exocytotic imaging with SRB. The two-photon excited fluorescence images of CFP and YFP (from YC-Nano15) and SRB were captured in separate channels (Channel 1, 2, and 4) through dichroic mirrors and emission filters, BP460-500, DM505/BP520-560 and DM570/BP575-630, respectively. Excitation laser power and high-voltage (HV) values for each channel were adjusted to obtain images with good signal-to-noise ratios and nearly equal intensities for CFP, YFP, and SRB under unstimulated conditions. The image acquisition speed was approximately 1 frame/s.

### 3.7. FRET Ratio Imaging Analysis

The public domain software ImageJ [19] was employed for image processing and calculations. Pixel intensities of the YFP image were divided by those of the CFP image to obtain the FRET (YFP/CFP) ratio image. Time-sequence images were aligned using the StackReg plugin [20] to compensate for horizontal movement during image acquisition. To calibrate the YC-Nano15 indicator, regions of interest (ROIs) with a diameter of 5 µm were chosen at the apical and basal sides of a single cell. Averages of the ratio value were calculated for each ROI. The ratio value was normalized to be 1 under normal conditions (*i.e.*, before stimulation). FRET signal images were represented as pseudo color images.

## 4. Conclusions

Here we report on establishment and evaluation of transgenic mice which have an ultrasensitive calcium indicator in their tissues and organs to visualize spatiotemporal dynamics of intracellular free calcium *in vivo* and *in vitro*. GECIs have been developed and widely used for live-cell calcium imaging, but they are not suitable for observation in some cells due to lack of sensitivity. In this study, we generated healthy transgenic mice ubiquitously expressing ultrasensitive GECIs and demonstrated the usefulness of these new mouse strains. Specifically, we performed FRET ratio imaging by two-photon microscopy of acinar cells in order to visualize calcium dynamics during minimal agonist stimulation. The results provide a new practical animal model for ultrasensitive calcium imaging *in vivo* or *in vitro* that could be widely utilized in biological and medical imaging analyses in the near future.

## Supplementary Materials

Supplementary materials can be found at http://www.mdpi.com/1422-0067/15/11/19971/s1.
